# Biodiversity of yeasts isolated during spontaneous fermentation of cool climate grape musts

**DOI:** 10.1007/s00203-020-02014-7

**Published:** 2020-08-11

**Authors:** Monika Cioch-Skoneczny, Paweł Satora, Szymon Skoneczny, Magdalena Skotniczny

**Affiliations:** 1 Department of Fermentation Technology and Microbiology, University of Agriculture in Krakow, ul. Balicka 122, 30-149, Krakow, Poland; 2 Department of Chemical and Process Engineering, Faculty of Chemical Engineering and Technology, Cracow University of Technology, Warszawska 24, 31-155 Krakow, Poland

**Keywords:** Non-*Saccharomyces*, Spontaneous fermentation, Cool climate, RAPD-PCR, 5.8S-ITS rRNA gene

## Abstract

Biodiversity of native yeasts, especially in winemaking, has hidden potential. In order to use the value of non-*Saccharomyces* strains in wine production and to minimise the possibility of its deterioration, it is necessary to thoroughly study the yeast cultures present on grape fruits and in grape must, as well as their metabolic properties. The aim of the study was to characterise the yeast microbiota found during spontaneous fermentation of grape musts obtained from grape varieties ‘Rondo’, ‘Regent’ and ‘Johanniter’. Grapes from two vineyards (Srebrna Góra and Zadora) located in southern Poland were used for the research. Succession of subsequent groups of yeasts was observed during the process. *Metschnikowia pulcherrima* yeasts were identified both at the beginning and the end of the process. *Hanseniaspora uvarum, Wickerhamomyces onychis* and *Torulaspora delbrueckii* strains were also identified during the fermentation. *Torulaspora delbrueckii* and *Wickerhamomyces onychis* strains were identified only in grape musts obtained from grapes of the Zadora vineyard. These strains may be characteristic of this vineyard and shape the identity of wines formed in it. Our research has provided specific knowledge on the biodiversity of yeast cultures on grapes and during their spontaneous fermentation. The research results presented indicate the possibility of using native strains for fermentation of grape musts, allowing to obtain a product with favourable chemical composition and sensory profile.

## Introduction

Grape must is a rich environment for various yeast species. Knowledge of the kinetics of their growth and metabolism is fundamental for understanding the influence of these microorganisms on the quality of wine. Fermentation process is related to several mechanisms, including metabolism of sugars and nitrogen compounds contained in grape must, enzymatic hydrolysis of grape components, yeast cell autolysis and bioadsorption of individual must components. All these factors determine the formation of a large number of compounds from the group of organic acids, higher alcohols, aldehydes or ketones, esters, glycerol responsible for the taste and smell of wine (Swiegers et al. 2005).

In the early stages of fermentation, the amount of non-*Saccharomcyes* yeast ranges from 10^3^–10^5^ to 10^6^–10^7^ CFU/mL (Hierro et al. 2006; Zott et al. 2008). Studies show high biodiversity of yeast microorganisms during the first 24–72 h of the process (Zott et al. 2008; Ocón et al. 2010). Factors such as mechanical damage of berries, agronomic practices and terroir (soil type, average annual temperature, rainfall) directly affect the biota of yeasts present on the surface of grapes (Barata et al. 2012; Díaz et al. 2013; Drumonde-Neves et al. 2016; Grangeteau et al. 2017).

Quantitative and qualitative characteristics of yeasts present on the grapes surface, must and wine were determined in numerous studies. It was found that the complexity of yeast microbiota during spontaneous grape must fermentation has a significant influence on the organoleptic and sensory properties of wine (Jolly et al. 2014; Padilla et al. 2016; Varela and Borneman 2017). Species from the genus *Hanseniaspora*, *Candida*, *Pichia*, *Zygosaccharomyces* and *Kluyveromyces* most desirably determine the diversity and complexity of the taste of wine (Romano et al. 2003; Jolly et al. 2014).

According to the decision of European Council of 20 December 2005, Poland was entered into so-called A zone of wine growing (the coldest one), referred to as ‘cool climate’ zone. Despite increasing temperatures caused by climate changes, conditions for growing vines in Poland are much less propitious than in traditional wine regions. Due to climatic and soil conditions, the obtained grapes are characterised by a lower content of sugars (usually 17–23%) and thus a low level of alcohol as well as higher acidity (Lisek 2011). However, this has its advantages in the form of a better balance between the content of sugar, acid and the pH value, as well as better cumulation of some aromatic compounds. Thanks to this, the cool climate wines can achieve very good quality. Higher acidity gives a sense of freshness, especially in the case of white wines (Sluys 2006). The grape varieties currently grown in Poland are characteristic of the cool climate region. Detailed research on the microbiota of grapes and grape must allow the identification of yeast strains characteristic to a specific terroir and defining the ‘identity’ of a regional wine.

The aim of the study was to characterise the yeast microbiota found during spontaneous fermentation of grape musts obtained from cool climate grape varieties ‘Rondo’, ‘Regent’ and ‘Johanniter’.

## Materials and methods

### Grapes and spontaneous fermentation of musts

Grapes of two red grape vine varieties (‘Rondo’, ‘Regent’) and one white grape variety (‘Johanniter’) from two vineyards located in southern Poland (Srebrna Góra—50° 2′ N, 19° 50′ E, and Zadora—49° 53′ N, 21° 52′ E) during two consecutive vintages (2013 and 2014) were taken in account of the study (Table 1).

**Table 1:** Grape varieties used in the study and harvest dates of grapes

Grape variety	Vineyard	
	Srebrna Góra	Zadora
Rondo	–	25.09.2013 28.09.2014
Regent	**–**	25.09.2013 28.09.2014
Johanniter	8.10.2013 4.10.2014	25.09.2013 28.09.2014

Ten bunches of mature grapes were gathered from several grape vines within a sub-area of each vineyard (100 m^2^). Then, berries were randomly selected (500 g), placed in sterile 500-mL flasks and pressed until juice has covered the fruits. The flasks were closed with airlocks filled with glycerine. Fermentation was carried out for 28 days at a temperature of 20 °C (each in triplicate).

### Physicochemical characteristics of grape musts

The analyses of pH, total acidity and sugar content were performed in accordance with the methodology described by Cioch-Skoneczny et al. (2018).

### Yeasts enumeration and isolation, DNA extraction and RAPD-PCR analysis, amplification of the 5.8S-ITS rRNA gene region, PCR–RFLP analysis

The analyses were performed in accordance with the methodology described by Cioch-Skoneczny et al. (Cioch-Skoneczny et al. 2018).

### 5.8S-ITS rRNA gene region sequencing

Amplified product of the rRNA gene was purified using Clean up AX (A&A Biotechnology, Poland) according to the manufacturer's instruction and submitted for sequencing to Macrogen Inc. (Netherlands). Species identification was achieved by comparing processed sequences with available in the GenBank database using the basic local alignment search tool (BLAST) at the https://www.ncbi.nlm.nih.gov/BLAST/. Percent homology scores were generated to identify yeast isolates. Sequences were deposited in the GenBank NCBI database with the accession numbers: MG971248 (*Torulaspora delbrueckii),* MG971245, MG971262 and MG971256 (*Metschnikowia pulcherrima*), MG971254 and MG971266 (*Hanseniaspora uvarum*), MG971267 (*Candida railenensis*), MH020215 (*Saccharomyces cerevisiae*) and MG971246 (*Wickerhamomyces onychis*).

## Results and discussion

### Kinetics of yeast population

**Fig. 1:**
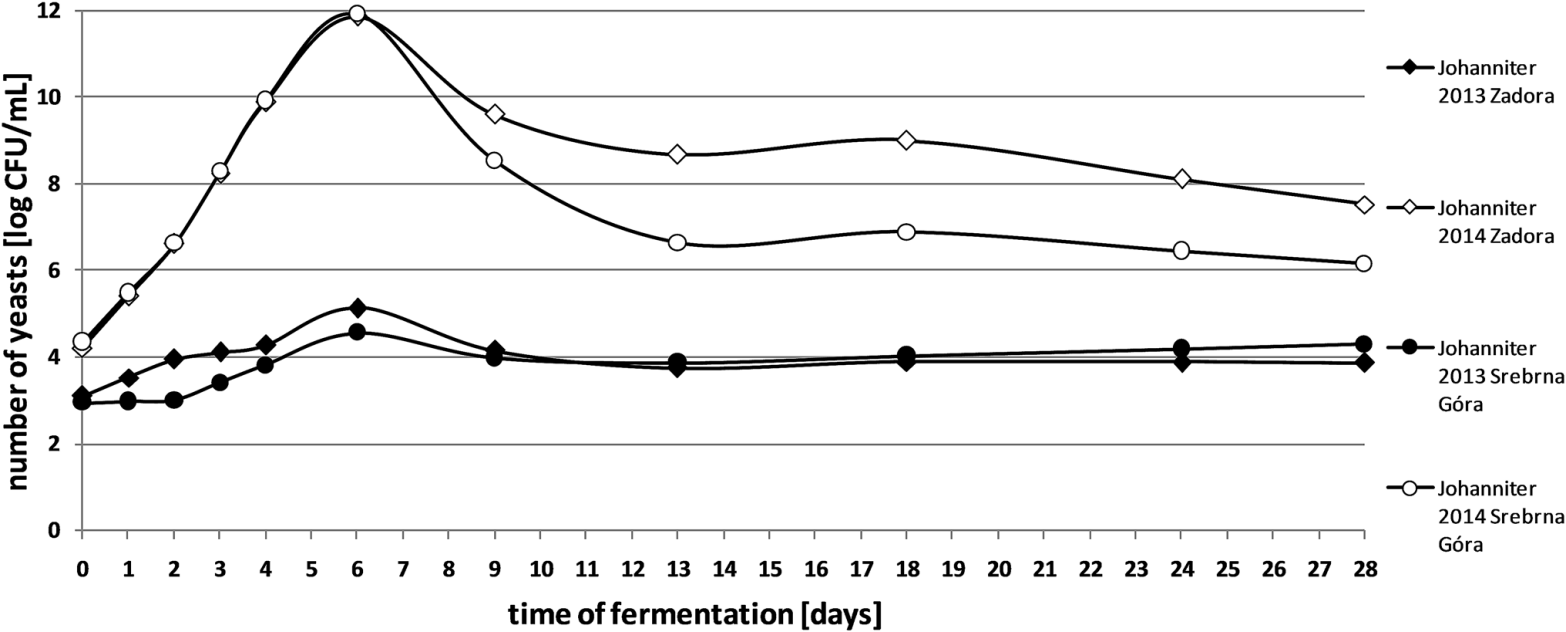
Quantitative microbiota of yeasts in spontaneously fermented grape musts obtained from Johanniter variety

**Fig. 2:**
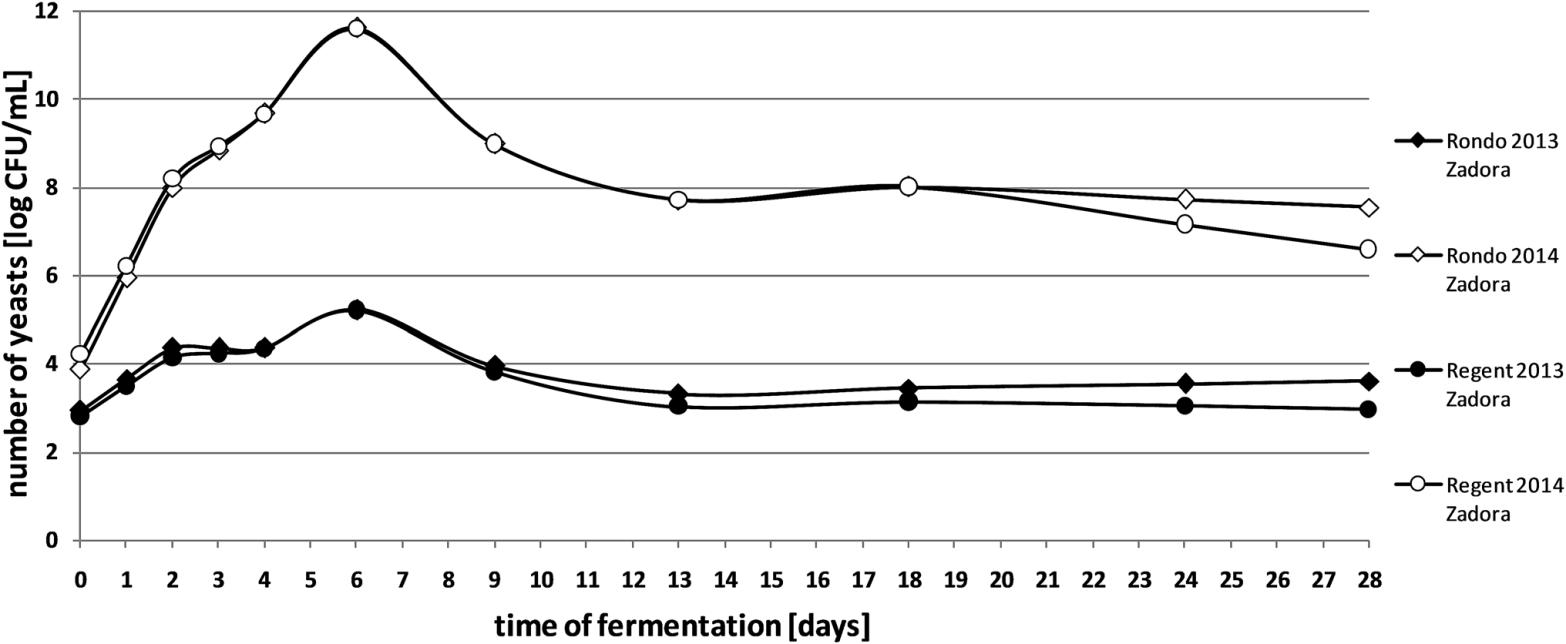
Quantitative microbiota of yeasts in spontaneously fermented grape musts obtained from Rondo and Regent varieties

The number of yeasts during spontaneous fermentation of grape musts remained at a similar level for all analysed grape varieties in 2014. In 2013, there were a smaller number of yeasts, in average by 4–5 logarithmic rows (Figs. 1 and 2). It could be caused by a small amount of food resources present in the fermenting medium in 2013, which probably limited the growth of microorganisms. Late spring frosts and early in the fruit ripening period, cooling and rain during flowering of the vine and additionally, the excess of rainfall in the summer season certainly influenced the quality of the grapes and the quantitative microbiota composition. According to literature data, the number of yeasts in fresh grape must varies in a wide range from 10^3^ to 10^7^ CFU/ mL (Hierro et al. 2006; Zott et al. 2008). This differentiation depends mainly on the degree of maturity, chemical composition and mechanical damage of fruits. The content of sugar and water in grapes is also significant, as well as pH value of must (Swiegers et al. 2005).

In 2014, in the first days of fermentation, a rapid increase in the yeast population in all analysed settings was observed. In 2013, this increase was slight (Figs. 1 and 2). In the initial phase of spontaneous fermentation mainly aerobic strains are developing, not very resistant to elevated alcohol concentration (Hornsey 2007).

A maximum of cells number in the grape musts was noted from the 6th to the 13th day of the process. The fast growth of yeasts at this stage of fermentation is confirmed by other studies (Romano et al. 2003).

After reaching the maximum of cells in grape musts, the number of microorganisms gradually decreased. This tendency continued until the end of the fermentation process in the batches obtained from the fruits from both vineyards (2014) (Figs. 1 and 2). Most likely, it was the result of the dieback of yeast species sensitive to the increasing concentration of alcohol, as well as the depletion of nutrients contained in the must and the accumulation of metabolites having an inhibitory effect on the growth of microorganisms. Literature data indicate that components produced during fermentation, such as fatty acids and ethanol, may also act as inhibitors that slow cell uptake of nitrogen. In addition, changing conditions, including oxygenation and clarification of must, have an undesirable effect on yeast growth and kinetics of the whole process (Ribéreau-Gayon et al. 2006). In the 2013 season, a slight increase in the number of microorganisms was noted at the end of spontaneous fermentation. At this stage of the process, strains of the genus *Saccharomyces*, resistant to higher concentrations of alcohol, predominate (Jolly et al. 2014).

**Fig. 3:**
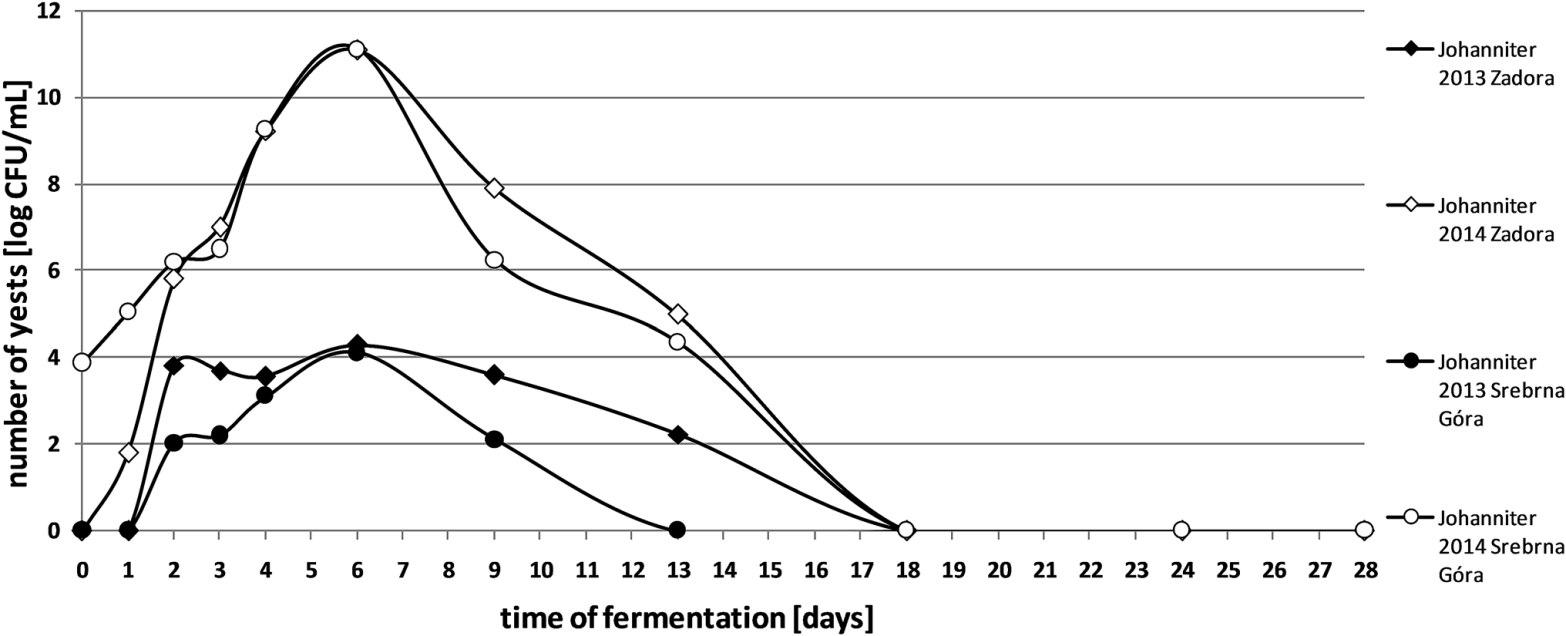
Changes in the number of *Kloeckera/Hanseniaspora* yeasts in spontaneously fermented grape musts obtained from Johanniter variety

**Fig. 4:**
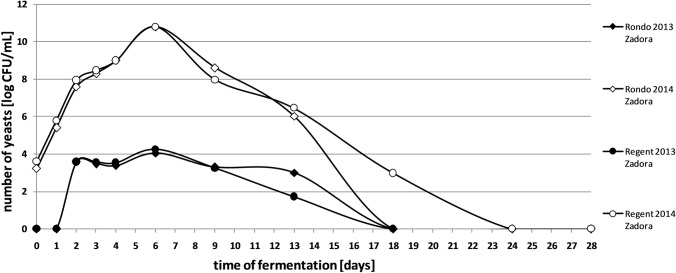
Changes in the number of *Kloeckera/Hanseniaspora* yeasts in spontaneously fermented grape musts obtained from Rondo and Regent varieties

Apart from determining the total yeast content, cultures on the WL medium allowed us to study the number of *Kloeckera*/*Hanseniaspora* sp. yeasts during spontaneous fermentation. Their content in fresh juices from ‘Rondo’ variety in 2014 season was 3.23 × 10^3^ CFU/mL. A comparable number of microorganisms was observed in fresh juices obtained from the ‘Regent’ and ‘Johanniter’ varieties (Srebrna Góra vineyard). These microorganisms were not recorded in fresh musts of the ‘Johanniter’ variety from Zadora vineyard. A similar dependence was found in 2013 season for all of analysed samples (Figs. 3 and 4). Studies show that some species belonging to the genus *Kloeckera*/*Hanseniaspora* show the ability to grow only under anaerobic conditions (Van de Water and Napa 2009). This may be the reason of missing them in the early days of fermentation in 2013 season.

The maximum yeast population in spontaneously fermented grape musts was recorded from the 5th to the 8th day of the process. While *Kloeckera*/*Hanseniaspora* species are the dominant microbiota in the first days of spontaneous fermentation, *Saccharomyces* strains are present in virtually undetectable quantities (Romano et al. 2003). In 2013, at this stage of fermentation, almost all detected yeasts belonged to the genus *Kloeckera*/*Hanseniaspora* (Figs. 1, 2, 3 and 4). Certainly, they did not actually represent all yeast present in the must, but their large numbers made the probability of isolation of these microorganisms high. This group of yeasts constituted even up to 95% of all microorganisms present in the batches. Literature results confirm such a high share of *Kloeckera*/*Hanseniaspora* yeast in grape musts at early stages of fermentation, which may amount to 99% of the entire microbiota (Ribéreau-Gayon et al. 2006).

Afterwards, the number of *Kloeckera*/*Hanseniaspora* cells in the analysed musts began to gradually decreased. From the 24th day of spontaneous fermentation, the presence of these cultures in the batches was not recorded (Figs. 3 and 4). Literature data report that some of this species can survive up to the last stages of the fermentation. In addition, these yeasts show tolerance to high concentrations of SO_2_ and low temperatures (Van de Water and Napa 2009). Selective use of fructose by some *Kloeckera*/*Hanseniaspora* species improves saccharide utilisation by *Saccharomyces* yeast, by reducing the risk of occurrence of the residual sugars after fermentation (Ciani and Fatichenti 1999).

### Physicochemical characteristics of grape musts

Depending on the variety, grape musts were characterised by a different acidity and sugar content (Table 2). Concentration of total sugars was quite similar within the varieties and ranged from 164.17 g/L (in ‘Rondo’ musts from Zadora 2014) to 223.80 g/L (in ‘Johanniter’ musts from Srebrna Góra 2013). In 2014 the concentrations of total sugars in grapes were lower than in the 2013 season (Table 2). ‘Johanniter’ must (Zadora 2014) characterised also relatively high total acidity (11.01 g/L).

**Table 2:** Characteristics of grape musts obtained from the Rondo, Regent and Johanniter grape varieties

Season	Vineyard	Grape variety	pH	Total acidity [g/L]^a^	Sugars [g/L]
2013	Srebrna Góra	Johanniter	3.12c (± 0.00)	8.40g (± 0.00)	223.80c (± 5.51)
2014	Srebrna Góra		3.19a (± 0.01)	8.03d (± 0.00)	204.17a (± 8.04)
2013	Zadora		3.06b (± 0.01)	8.20f (± 0.00)	214.00ac (± 3.12)
2014	Zadora		3.20a (± 0.02)	11.01a (± 0.00)	166.67b (± 2.89)
2013	Zadora	Rondo	3.06b (± 0.01)	8.13e (± 0.02)	201.50a (± 5.63)
2014	Zadora		3.11c (± 0.01)	9.89h (± 0.00)	164.17b (± 3.82)
2013	Zadora	Regent	3.21a (± 0.02)	6.72b (± 0.01)	205.83a (± 6.29)
2014	Zadora		3.31d (± 0.07)	7.28c (± 0.01)	165.00b (± 8.66)

The results marked with the same letters do not differ significantly (*p* > 0.05)

^a^Expressed in g/L of malic acid

### Yeasts identification

In total, 162 (in 2013) and 78 (in 2014) pure yeast cultures were isolated from various stages of fermented musts three varieties of grapes.

Isolates were typed by RAPD-PCR in order to characterise the identical biotypes and to reduce the number of samples taken for further analysis. All isolates were classified into groups characterised by distinct electrophoretic patterns.

Representatives of each groups of RAPD patterns were analysed by 5.8S-ITS PCR–RFLP. Ultimately, representatives of each groups of RFLP patterns were identified by 5.8S-ITS rRNA gene region sequencing.

In 2013 and 2014 seven different yeast species were distinguished. According to the sequencing results, the most identified strains belonged to the species *Metschnikowia pulcherrima, Wickerhamomyces onychis, Torulaspora delbrueckii, Candida railenensis*, *Saccharomyces cerevisiae* and *Hanseniaspora uvarum* (Table 3).

**Table 3:** Identified yeast species on the basis of their lengths of restriction fragments of the 5.8S-ITS rRNA gene region and the highest 5.8S-ITS rRNA similarity score

5.8S-ITS [bp]	Restriction fragments [bp]			Species identification (% identity)*	Accession no
	*Hinf I*	*Hae III*	*Cfo I*		
650	350 + 180 + 150	650	300	*Candida railenensis* (99%)^1^	MG971267
750	350 + 190	390 + 290 + 180 + 120	320 + 200 + 170 + 100	*Hanseniaspora uvarum* (98%)	MG971254
750	320	380 + 220	750	*Hanseniaspora uvarum* (98%)	MG971266
390	200	300	210 + 100	*Metschnikowia pulcherrima* (99%)	MG971245
390	350 + 200	300 + 200	200 + 180	*Metschnikowia pulcherrima* (99%)	MG971256
390	200	280 + 180	210 + 180 + 100	*Metschnikowia pulcherrima* (98%)	MG971262
880	400	350 + 250 + 200 + 150	350	*Saccharomyces cerevisiae* (98%)^2^	MH020215
800	400 + 200	800	350 + 250 + 150	*Torulaspora delbrueckii* (98%)	MG971248
600/650	350 + 200	650 + 500 + 350	450 + 350 + 180	*Wickerhamomyces onychis* (98%)^3^	MG971246

*According to BlastN search of 5.8S-ITS rRNA gene sequences in NCBI database

^1^Isolated from spontaneously fermented musts of Rondo and Regent grape varieties (Spotkaniówka and Srebrna Góra vineyard)

^2^Isolated from spontaneously fermented musts of Rondo and Bianca grape varieties (Spotkaniówka vineyard), Hibernal variety (Spotkaniówka and Srebrna Góra vineyard) and Seyval Blanc variety (Srebrna Góra vineyard)

^3^Isolated from spontaneously fermented musts of Seyval Blanc grape variety (Zalipie vineyard)

Tables 4, 5, 6 and 7 present the percentage distribution of yeast strains isolated from different stages of spontaneous fermentation of grape musts obtained from ‘Rondo’, ‘Regent’ and ‘Johanniter’ varieties in two consecutive years. The cultures of *M. pulcherrima* dominated. They were identified at each stage of spontaneous fermentation of grape musts. *W. onychis* and *T. delbrueckii* species were isolated from the Zadora vineyard from fermented ‘Johanniter’ and ‘Regent’ musts.

**Table 4:** Distribution of yeast strains (%) isolated from different stages of the Johanniter (Zadora) grape musts spontaneous fermentation (2013 and 2014 season)

Strain	Accession no	2013											2014										
		Sampling day											Sampling day										
		0	1	2	3	4	6	9	13	18	24	28	0	1	2	3	4	6	9	13	18	24	28
*Candida railenensis*	MG971267		10		10			10		10			10	10	20	10	20	10		20	10	10	
*Hanseniaspora uvarum*	MG971254	20	30	30	20	40	50	50	40				10	15	20	10	15	20	50	30			
*Hanseniaspora uvarum*	MG971266	30	20	10	20	20	20	10	20				20	15	30	10	25	30	20	10			
*Metschnikowia pulcherrima*	MG971245																						
*Metschnikowia pulcherrima*	MG971256	20	10	30		10	20		20	30	20	10	20	10	10	10	20	10		40	30	10	
*Metschnikowia pulcherrima*	MG971262	20	10	30	30	20		20		20	20	10	20	30	20	30	20	20	20		20	20	
*Saccharomyces cerevisiae*	MH020215									30	60	80									40	60	100
*Torulaspora delbrueckii*	MG971248		10		10		10		20				10	20		20							
*Wickerhamomyces onychis*	MG971246	10	10		10	10		10		10			10			10			10

**Table 5:** Distribution of yeast strains (%) isolated from different stages of the Johanniter (Srebrna Góra) grape musts spontaneous fermentation (2013 and 2014 season)

Strain	Accession no	2013											2014										
		Sampling day											Sampling day										
		0	1	2	3	4	6	9	13	18	24	28	0	1	2	3	4	6	9	13	18	24	28
*Candida railenensis*	MG971267	20	15	20	20	20	15		10	20	10		20	10	20		20	10		10			
*Hanseniaspora uvarum*	MG971254																						
*Hanseniaspora uvarum*	MG971266	30	25	30	20	40	50	60					40	30	20	40	30	50	50	20			
*Metschnikowia pulcherrima*	MG971245																						
*Metschnikowia pulcherrima*	MG971256	30	30	30	40	20	25	20	30	20	20	10	10	20	40	30	25	20	30	40			
*Metschnikowia pulcherrima*	MG971262	20	30	20	20	20	10	20	40	20	20		30	40	20	30	25	20	20	30	40	60	
*Saccharomyces cerevisiae*	MH020215								20	40	50	90									60	40	100
*Torulaspora delbrueckii*	MG971248																						
*Wickerhamomyces onychis*	MG971246

**Table 6:** Distribution of yeast strains (%) isolated from different stages of the Rondo (Zadora) grape musts spontaneous fermentation (2013 and 2014 season)

Strain	Accession no	2013											2014										
		Sampling day											Sampling day										
		0	1	2	3	4	6	9	13	18	24	28	0	1	2	3	4	6	9	13	18	24	28
*Candida railenensis*	MG971267	10	20	10		20		20		10			20	10	20	30	15		20	20	30	15	
*Hanseniaspora uvarum*	MG971254	20	15	20	20	40	50	50	10				20	30	10	25	15	50	60	10			
*Hanseniaspora uvarum*	MG971266	30	25	10	10		20	15	20				30	15	20	15	40	30	10	15			
*Metschnikowia pulcherrima*	MG971245	20	10	20	20	10		15		20	20	10	10	30	30	15	30	20	10	55	50	40	10
*Metschnikowia pulcherrima*	MG971256		20	20	40	30	30		20	40	20	10											
*Metschnikowia pulcherrima*	MG971262	20	10	20	20				50	10			20	15	20	15							
*Saccharomyces cerevisiae*	MH020215									20	60	80									20	45	90
*Torulaspora delbrueckii*	MG971248																						
*Wickerhamomyces onychis*	MG971246

**Table 7:** Distribution of yeast strains (%) isolated from different stages of the Regent (Zadora) grape musts spontaneous fermentation (2013 and 2014 season)

Strain	Accession no	2013											2014										
		Sampling day											Sampling day										
		0	1	2	3	4	6	9	13	18	24	28	0	1	2	3	4	6	9	13	18	24	28
*Candida railenensis*	MG971267	10	20	20	30	20		10	10					10	20	20	10	10	10	10			
*Hanseniaspora uvarum*	MG971254	10	15	20	10	20	40	40	20				20	30	10	20	30	40	50	20			
*Hanseniaspora uvarum*	MG971266	20	15	20	20	30	40	20	10				25	20	20	20	10	30	20	10			
*Metschnikowia pulcherrima*	MG971245	10	20	10		20		10	20	10	10		15	10	15		15		10	20	40	50	
*Metschnikowia pulcherrima*	MG971256	20							20	30			15	20	15	10							
*Metschnikowia pulcherrima*	MG971262	10	20	20	20		10	20		20	30	10	15	10	10	30	25	10	10	40	60		
*Saccharomyces cerevisiae*	MH020215									40	60	90										50	100
*Torulaspora delbrueckii*	MG971248	10		10		10																	
*Wickerhamomyces onychis*	MG971246	10	10		20		10		10				10		10		10	10					

Most of the identified microorganisms belonged to the genus *Kloeckera*/*Hanseniaspora* (described above) and *M. pulcherrima* species. These yeasts occurred frequently during the entire fermentation process in settings from both vineyards (Tables 4, 5, 6 and 7). However, the *M. pulcherrima* strain (MG971245) was not registered in grape musts and wines obtained from the white grape ‘Johanniter’ variety. Bisson and Joseph (Bisson and Joseph 2009) showed a high participation of strains, among others from *Metschnikowia* genus, on the surface of ripe grapes. Literature data indicate a decrease in the number of these cultures in grape must after 100–130 h of spontaneous fermentation and their absence after 10 days of the process (Cocolin et al. 2000). In turn tests carried out by Díaz et al. (2013) have proved the occurrence of *M. pulcherrima* strains in fermenting grape musts at least 5 days longer. Medina et al. (2012) have reported that *Hanseniaspora viniae* and *M. pulcherrima* strains can use enough nutrients, contributing to slow down fermentation.

*Candida railenensis* strain was identified in all fermented grape musts. It was not detected in the final stage of spontaneous fermentation, which is confirmed by our earlier research (Cioch-Skoneczny et al. 2018). These species dominate in the early stages of the process (Jolly et al. 2006; Bagheri et al. 2015).

*Wickerhamomyces onychis* strain was identified in fermented grape musts obtained from the ‘Regent’ and ‘Johanniter’ varieties (Zadora vineyard) (Tables 4 and 7). There is little information on the occurrence of this species in grape musts. However, it is known that strains belonging to the genus *Wickerhamomyces* may persist in must until the end of the fermentation process (Díaz et al. 2013). Some of them tolerate up to 12.5% (v/v) of ethanol. They are also able to produce killer toxins (Walker 2011; Sabel et al. 2014), which allowing them to compete with other yeasts in the same environment. However, in fermenting musts, their growth is limited due to the lack of oxygen (Walker 2011). Numerous studies have proved the occurrence of *W. anomalus* strains in fermented grape musts (de Ponzzes-Gomes et al. 2014; Bagheri et al. 2015). These yeasts were identified in musts obtained from Polish white grape variety—‘Hibernal’ (Cioch-Skoneczny et al. 2019). The presence of these strains in cultures mixed with *S. cerevisiae* strains contributes to the improvement of the aroma of wines (Izquierdo Cañas et al. 2014).

In recent years, the use of non-*Saccharomyces* yeast for the production of industrial wine has been considered (Romano et al. 2003; Suárez-Lepe and Morata 2012). Studies show that some species, such as *M. pulcherrima*, *Torulaspora delbrueckii*, *Hanseniaspora uvarum*, *Rhodoturula mucillaginosa*, *Pichia kluyveri* or *Candida* spp. used in monocultures or cultures mixed with *S. cerevisiae*, can improve the taste characteristics of wine (Gobbi et al. 2013; Belda et al. 2015). *T. delbrueckii* species shows special properties. Controlled inoculation of this yeast is recommended to improve the complexity and enhancement of wine traits (Jolly et al. 2006; Azzolini et al. 2012). Their presence contributes to the increase of glycerol in wine (Contreras et al. 2014) and mannoproteins (Belda et al. 2015), as well as to the reduction of alcohol content (Contreras et al. 2014). It has been proven that mixed with *S. cerevisiae* strains, they can contribute to the reduction of volatile acidity, acetoin and acetaldehyde levels (Velázquez et al. 2015), leading to growth content of 2-phenylethanol, terpinenol and lactones in wine (Azzolini et al. 2012; Velázquez et al. 2015). *T. delbrueckii* yeast was not detected in spontaneously fermented grape musts of ‘Johanniter’ variety obtained from the Srebrna Góra vineyard (Table 5).

*Saccharomyces cerevisiae* yeast are one of the dominant cultures present during spontaneous must fermentation, responsible for the chemical and sensory properties of wine (Romano et al. 2003; Camarasa et al. 2011). Numerous studies based on the analysis of DNA polymorphism indicate a large genetic diversity of this species (Capece et al. 2013). Despite the appearance of a significant number of different strains of *S. cerevisiae* at the beginning of fermentation, only a few (from one to three) predominate in the final stage (Capece et al. 2016). Research conducted by Knight et al*.* (2015) concerning the biogeographic characterisation of *S. cerevisiae* wine yeasts revealed the presence of a regional population with a specific genotype, but without differentiation within the region. These experiments suggest that specific, native strains may be associated with terroir and determine the typical nature of wines (Van Leeuwen and Seguin 2006; Capece et al. 2016). In the analysed grape must, one strain of *S. cerevisiae* (Tables 4, 5, 6 and 7) was detected, which dominated at the end of the spontaneous fermentation step. On the last day of the process, the number of identified isolates reached nearly 100%.

## Conclusions

Grape musts obtained from fruits of the red grape varieties ‘Rondo’, ‘Regent’ and white variety ‘Johanniter’ proved to be a very good environment for yeast growth. In comparison with 2014, the 2013 season was characterised by a smaller content of microorganisms in fresh grape juice, which resulted in their quantity during spontaneous fermentation. The highest number of microorganisms constituted *Hanseniaspora* strains, which, after the 13th day of fermentation, were replaced by *Saccharomyces* cultures. *Torulaspora delbrueckii* and *Wickerhamomyces onychis* strains were identified only in grape musts obtained from fruits from the Zadora vineyard. These strains may be characteristic of this vineyard and shape the identity of wines formed in it.
